# Common variants in glucuronidation enzymes and membrane transporters as potential risk factors for colorectal cancer: a case control study

**DOI:** 10.1186/s12885-017-3728-0

**Published:** 2017-12-28

**Authors:** Sabrina Falkowski, Jean-Baptiste Woillard, Deborah Postil, Nicole Tubiana-Mathieu, Eric Terrebonne, Antoine Pariente, Denis Smith, Rosine Guimbaud, Claire Thalamas, Koukeb Rouguieg-Malki, Pierre Marquet, Nicolas Picard

**Affiliations:** 1 Oncology Department, CHU Limoges, Limoges, France; 2 UMR-1248, Inserm, CHU Limoges, Univ. Limoges, Limoges, France; 3 Clinical Investigation Centre, CHU Limoges, Limoges, France; 4 Hepato-gastro-enterology Department, University Hospital Bordeaux, Bordeaux, France; 5 Pharmaco-epidemiology unit, University Hospital Bordeaux, Bordeaux, France; 6 Oncology Department, University Hospital Bordeaux, Bordeaux, France; 7 Digestive Oncology Department, University Hospital Toulouse, Toulouse, France; 8 Clinical Investigation Centre, University Hospital Toulouse, Toulouse, France; 9 Service de Pharmacologie, Toxicologie et Pharmacovigilance, CHU Limoges, Bâtiment CBRS, 2 avenue Martin-Luther King, F-87042 Limoges, France

**Keywords:** Colorectal cancer, Pharmacogenetics, UGT, Efflux transporters, OATP

## Abstract

**Background:**

Associations between polymorphisms of UDP-glucuronosyltransferases (UGTs) or efflux transporters (e.g., P-glycoprotein and MRP2) and different types of cancer have been described, whereas the role of influx transporters (e.g. OATP1B1 and OATP2B1) has been seldom explored. The GenColon study investigated potential associations between variant alleles of UGTs, efflux and influx transporters and CRC.

**Methods:**

Three hundred CRC cases were matched with 300 controls for age, sex and enrolment site. Fifteen SNPs in *UGT1A6–9, UGT2B7, ABCB1, ABCC2, SLCO1B1* and *SLCO2B1* genes were characterized using Taqman® PCR. Using multivariate conditional logistic regression, we investigated the relationships between CRC and “environmental” risk factors (physical activity, housing and working areas, consumption of red meat, tobacco, alcohol); genetic polymorphisms, in the study population and in the subgroups with “environmental” risk factors.

**Results:**

No significant association was observed for the analyzed SNPs (or haplotypes). However, an increased CRC risk was found in carriers of the *UGT1A8* rs1042597-G variant allele (additive risk OR = 3.39[1.29–8.89], *p* = 0.02951) in the subgroup of meat-consumers (*n* = 84), and in carriers of the *ABCB1* rs1045642-T (exon26) variant allele (additive risk; OR = 1.89[1.10–3.39], *p* = 0.0257) in the “never alcohol consumption subgroup” (*n* = 125). In addition, as previously reported, the following CRC risk factors were identified: absence of physical activity (OR = 6.35[3.70–10.9], *p* < 0.0001), living or working in rural or mix area (OR = 2.50[1.48–4.23], *p* = 0.0006 and OR = 2.99[1.63–5.48], *p* = 0.004, respectively) and tobacco exposure >30 years (3.37[1.63–6.96], *p* = 0.0010).

**Conclusions:**

Variant genotypes of influx transporters (OATP1B1 and 2B1) were not associated with CRC. This study confirmed the influence of lifestyle factors, but not the previously reported detrimental effect of SNPs in intestinal UGTs or efflux transporters, except for a *UGT1A8* variant in subjects consuming meat and the exon 26 SNP of ABCB1 in the never alcohol consumption subgroup.

**Trial registration:**

Registered in Direction Générale de la Santé the 1st July 2008 under the number DGS2008–0144.

**Electronic supplementary material:**

The online version of this article (10.1186/s12885-017-3728-0) contains supplementary material, which is available to authorized users.

## Background

Colorectal cancer (CRC) is the third and the second leading cause of cancer-related mortality in French male and female populations, respectively [1]**.** In the case of sporadic CRC, lifestyle risk factors play a pivotal role in the aetiology of the disease, including diet [2, 3], physical activity [4], obesity [5, 6], and cigarette smoking [7, 8]. A high consumption of red meat, cooking methods and alcohol drinking have also been associated with a higher risk of colorectal cancer [9–11]. Carcinogenic polycyclic aromatic hydrocarbons (PAH) and heterocyclic amines (HCA) originate from certain cooking methods, namely boiling, grilling or pan-frying which particularly influence meat doneness. Cigarette smoke also contains a variety of PAH and HCA. These compounds can be activated by phase I metabolic enzymes and detoxified by phase II enzymes, including UDP-glucuronosyltransferases (UGTs) [12] that glucuronidate HCA and PAH. Giuliani et al. reported that UGTs may participate in the early stage of colon malignant transformation and might be acted upon in the prevention against carcinogenesis [13]. Consumption of pan-fried red meat, UGT1A7 low-activity genotypes and UGT1A9 high/intermediate-activity genotypes were positively associated with the occurrence of CRC [14, 15]. Chronic inflammation of the intestine is another risk factor of CRC. Higher consumption of aspirin or NSAIDs, or aspirin use over longer periods may be associated with a reduction in the incidence of CRC [16]. The protective effect of aspirin would be influenced by the genetic polymorphisms of UGT1A6 and UGT2B7 [17–19]. Finally, polymorphic expression analysis of *UGT1A* genes in colon cancer did find that *UGT1A8* was up-regulated in the tumour compared with healthy tissue from the same patients [20].

The Permeability-glycoprotein (P-gp) (coded by the *ABCB1* gene) and the Multidrug Resistance-associated Protein 2 (coded by *ABCC2*) belong to a group of ATP-dependent efflux pumps and are abundant in the intestine. There has been evidence that polymorphisms of *ABCB1* affect P-gp activity and related expression [21, 22]. Specifically, the first study suggesting that *ABCB1* c.3435C > T has a significant impact on P-gp activity was published by Hoffmeyer et al. [23]. The authors showed that individuals with the homozygous genotypes had a lower ABCB1 expression in the intestine (approx. 2-fold decrease) and increased digoxin plasma levels (a typical P-gp substrate) as compared to individuals bearing the 3435CT or CC genotypes. Wang et al. confirmed that this particular variant was associated with decreased mRNA expression presumably by decreased stability [24]. Finally, Kimchi-Sarfaty et al. showed more recently that the variant altered the folding of the protein, hence its conformation and activity [25]. Several studies showed that three of the most frequent Single Nucleotide Polymorphisms (SNPs) of *ABCB1* (rs1128503, rs2032582, rs1045642) may impact the risk of cancer development, including CRC [26–29]. Specifically, a meta-analysis of case-control studies on 3175 cases and 3715 controls highlighted an increased frequency of the wild-type (rs2032582G/rs1045642C) combined allele (or haplotype) in Caucasian (but not Asian) patients with cancer (OR = 1.22, [95%CI][1.03–1.44],*p* = 0.02) [30]. Ewa Balcerczak et al. [31] analysed 95 tumour specimens and found a significant association between the third polymorphism (rs1128503 allele 1236-T) and CRC progression (HR = 0.26; *p* = 0.0424). Only a few studies of *ABCC2* polymorphisms as risk factors of CRC have been reported so far, and they have not evidenced any significant relationship [26, 32–34].

Organic anion-transporting polypeptides (OATPs) encoded by the *SLCO* genes are influx transporters working jointly with phase I- and II-metabolizing enzymes, as well as efflux transporters. This leads to a complex interplay between uptake, biotransformation and efflux of drugs, which strongly affects drug absorption as well as drug concentration in the intestinal cell lines [35, 36]. There has been increasing evidence that OATPs play an important role in the biology of various cancers [37]. A recent study highlighted the overexpression of OATP2B1 (known as a prostaglandin PGE2 transporter, coded by the *SLCO2B1* gene), which is substantially expressed in the intestine, in CRC biopsies (19 patients enrolled,11 in the neoplasia group and 8 in the control group) (*p* = 0.017) [38]. Indeed, OATP2B1 might be involved in chronic inflammatory processes which are known to be CRC risk factors. *SLCO1B1* mRNA was detected mainly in liver but also in enterocytes of the small intestine [39]. *SLCO1B1* c.521 T > C SNP (p.V174A, rs4149056) decreased the activity of transporter. In a Turkish case-control study (100 cases, 150 controls), this allelic variant was statistically associated with the susceptibility to CRC (OR = 2.66 [1.31–5.41], *p* = 0.0057) [40].

The aim of the present French, carefully paired case-control study was to re-evaluate and hierarchize all these potential genetic risk factors of CRC, namely polymorphisms in *UGT1A6–9, UGT2B7, ABCB1, ABCC2, SLCO1B1* and *SLCO2B1,* taking into account the main environmental risk factors as pairing criteria of stratification factors.

## Methods

### Subjects

The study population comprised 300 patients with CRC (cases) matched with 300 individuals without evident cancer. Cases and controls were included between February 2009 and October 2010. The controls were matched to cases according to sex, age, and recruitment site (among four University hospitals in France). In each hospital, the “clinical investigation center” recruited the controls and the oncology department the cases. The included cases were newly diagnosed histologically proven cases, whatever the disease state. Each participant answered a one-page standardized questionnaire on lifestyle and food habits (physical activity, meat consumption, tobacco exposure, alcohol consumption, working/housing environments and NSAID consumption). To be eligible, participants had: to be 18 years old or more; to be mentally and physically able to participate; and to sign a specific informed consent form for providing biological samples for genetic analysis. The participant with a family history of CRC, adenomatous polyposis, ulcerative colitis or Crohn’s disease was excluded because of a specific molecular profile suspected in familial cancer cases and high NSAID consumption or use of immunosuppressive therapy for the treatment of these chronic diseases. The local and national ethic committees (CPP du Sud Ouest et Outre-mer IV) approved the protocol the 12th february 2009 (#CPP-AC09–002). A written informed consent was obtained from each individual included in the study, after clear explanation of the research protocol by a physician. All information regarding participants was made anonymous.

### Selection and analysis of low-penetrance genes and allelic variants

Published studies were traced using Medline from 2000 to 2016 (until September), using the search terms colorectal cancer AND risk factors (i.e. tobacco, meat, cooking, physical activity, alcohol, aspirin), lifestyles, environmental factors, rurality, xenobiotics; then colorectal cancer AND UGT (and synonymous words), efflux transporters (and synonymous words), influx transporter (and synonymous words) polymorphism(s) to identify candidate genes. For each specific candidate gene, a separate search was performed. The selected candidate gene polymorphisms in *UGT*, *ABC* or *SLCO* were: (i) relevant variants associated with sporadic CRC and reported in the National Center for Biotechnology Information SNP database, with a minor allelic frequency (MAF) ≥10% in Caucasians; or (ii) variants in genes expressed in the intestine and reported in the literature with a convincing functional effect (i.e., with in vitro or in silico evidence). As a result, fifteen SNPs in *UGT1A6* (rs1105879), *UGT1A7* (rs11692021)*, UGT1A8* (rs1042597), *UGT1A9* (rs2741045), *UGT2B7* (rs7438135*), ABCB1* (rs1128503, rs2032582, rs1045642*), ABCC2* (rs717620, rs2273697, rs3740066)*, SLCO1B1* (rs4149056, rs2306283) and *SLCO2B1* (rs2306168, rs12422149) genes were selected. In order to make this study easier to read, we graded the quality-of-evidence (QOE) regarding the effect of genetic variants as retrieved in Pubmed, from which we derived using previously reported criteria [41] a level-of-recommendation (LOR), to eventually select ‘highly recommended’ candidates for further assessment as CRC risk factors (Table 1).

**Table 1:** Criteria used for grading the quality of evidence of candidate genetic variants related to the pharmacodynamic pathways of immunosuppressive drugs, and the level of recommendation for further research in occurrence of CRC

Grade	Definition
Quality of evidence	
A	Evidence from one or more well-designed clinical study, and a clear mechanistic rationale supported by at least one in vitro *or* ex vivo functional study
B	Evidence from ≥2 clinical studies with consistent data, and clear mechanistic rationale supported by in silico prediction (no in vitro functional study)
C	Evidence from ≥2 clinical studies with consistent data but unclear or no mechanistic basis
D	Conflicting data and convincing data against a functional effect
Level of recommendation for further research in CRC risk factor	
1	Highly recommended candidate: QOE of grade A or B, and ≥1 positive association study in CRC risk factor or candidate not yet studied in CRC with a QOE of grade A
2	Recommended candidate: candidate not yet studied in CRC risk factor with a QOE of grade B
3	Potential candidate: QOE of C or candidate with a QOE of grade A or B unsuccessfully tested in one CRC risk factor (≤2 negative studies)
4	Candidate to exclude: QOE of grade D or a QOE of grade A, B or C with convincing data against any statistical association in CRC risk factor (>2 negative studies)

Variants were not classified if no minor allele frequency was available in the dbSNP, or if evidence was strictly limited to a single in silico, in vitro, ex vivo or clinical study

QOE: Quality of evidence; CRC: Colorectal cancer

### Genotype analysis

Genomic DNA was isolated from whole blood samples using the QIAamp isolation system (Qiagen, Hilden, Germany). Concentrations were determined by UV absorption spectroscopy at 260 and 280 nm (Nanodrop, Labtech, France). Solutions of DNA at 2 ng/μL were analyzed using appropriate TaqMan real-time PCR discrimination assays (Life Technologies, Saint Aubin, France) to characterize the 15 different polymorphisms (Rotor-Gene, Qiagen, France). Primers were designed and synthesized by Life Technologies. The reaction mix consisted of 5 μL TaqMan Universal Master Mix, primers and probes, 10 ng of DNA template and water for a total reaction volume of 10 μL. Analyses were performed on an ABI 7000 real-time PCR system (Applied Biosystems) or a Rotorgene Q instrument (Qiagen) using the manufacturer protocol.

### Lifestyle variables

Variable were categorized following the WHO recommendations or the literature as red meat consumption (<3 portions/week vs. ≥ 3 portions/week) [9, 42], alcohol consumption (never vs. ≤30 g/day vs. > 30 g/day now or in the past) [43, 44], tobacco consumption (never vs. tobacco exposure <30 years vs. ≥ 30 years) [45] and physical activity (<30 min/day vs. ≥30 min/day) [9, 42]. Rural / urban housing and rural / urban workplace stratification was based on the ZIP code. The consumption of NSAID drugs could not been analyzed, due to too many missing data about drug names, doses and intake frequency, and sometimes confusion between analgesics and NSAIDs by the subjects.

### Statistical analysis

The statistical analyses were performed using R software version 3.1.1 (R foundation for statistical computing, http://www.r-project.org). All polymorphisms were tested for Hardy-Weinberg equilibrium in case and controls separately. The effect of SNP on CRC was investigated using an additive risk model (called 0, 1, 2 in which each allele confers an increased risk). A power calculation was performed using the gap R package: for 590 case-controls with an alpha risk = 0.05, a genotype relative risk of 2 with an additive genetic model, we have an 80% power to detect an effect of the genotype for frequencies equal or higher than 0.08. In this case only the analysis of *SLCO1B2* c.1457C > T (rs2306168) is underpowered (MAF = 0.02, power = 0.33).

Most probable *ABCB1* haplotype were inferred using the “SNPassoc” R package. For the Multivariate conditional logistic regression was used to investigate the effect of i) CRC and “environmental” risk factors (physical activity, housing and working conditions, consumption of red meat, tobacco, alcohol); ii) CRC and genetic polymorphisms in the whole study population; and iii) gene-environment interaction for CRC for tobacco, alcohol and meat consumption. In a first step, each covariate was tested in univariate analysis. In the second step, all the significant covariates characterized by a *p* < 0.1 in the univariate analysis were included simultaneously in an intermediate multivariate model, and a backward stepwise process using the Akaike criterion was applied to select the final model. Stability of the final models was validated by performing 1000 bootstraps followed by 1000 multivariate backward stepwise procedures. *P* values <0.05 were considered to be statistically significant.

## Results

Data from 295 patients with sporadic CRC (with or without metastasis) and 295 unaffected, paired controls were available for statistical analyses. Five case-control pairs were excluded because of technical problems or missing data regarding either the case or the control. The main characteristics of the two groups are presented in Table 2. Overall, the mean age was 66 years, gender mostly male (61%), and the mean body mass index was 26 kg/m^2^ (similar in both groups). There was no significant difference between the four investigating centers regarding patients’ and controls’ lifestyles.

**Table 2:** Demographics of the cases and controls

Demographic characteristics and known risk factors	Cases (*n* = 295)	Controls (*n* = 295)
Age (±SD)	66 (±11)	66 (±11)
Sex		
*M*	179	179
*F*	116	116
BMI (±SD)	26 (±4)	26 (±4)
Living		
*Rural / Mix*	159 *(53,9%)*	87 *(29,5%)*
*Urban*	136 *(46,1%)*	207 *(70,2%)*
Job		
*Rural/ Mix*	114 *(38,7%)*	47 *(15,9%)*
*Urban*	180 *(61%)*	247 *(83,7%)*
Red meat consumption		
*< 3 portions/week*	244 *(82,7%)*	261 *(88,5%)*
*≥ 3 portions/week*	51 *(17,3%)*	34 *(11,5%)*
Tobacco consumption		
*Never*	168 *(57%)*	174 *(59%)*
*Tobacco exposure < 30 years*	72 *(56,7%)*	88 *(72,7%)*
*Tobacco exposure ≥ 30 years*	55 *(43,3%)*	33 *(27,3%)*
*Cessation < 10 years*	33 *(26%)*	15 *(12,4%)*
*Cessation ≥ 10 years*	89 *(70,1%)*	106 *(87,6%)*
Alcohol consumption		
*Never*	85 *(28,8%)*	41 *(13,9%)*
*≤ 30 g/day*	163 *(77,6%)*	233 *(91,7%)*
*> 30 g/day now or in the past*	45 *(21,4%)*	21 *(8,3%)*
Physical activity		
*< 30 min/day*	207 *(70,2%)*	97 *(32,9%)*
*≥ 30 min/day*	87 *(29,5%)*	197 *(66,8%)*

### Association between lifestyle factors and CRC

Table 3 describes the influence of environmental factors on CRC obtained after univariate, multivariate analyses and after internal validation using bootstraps. In multivariate analysis, the absence of physical activity ((<30 min/day vs. ≥30 min/day, OR = 6.35[3.70–10.91], *p* < 0.0001), population with rural or mix housing (rural or mix vs. urban, OR = 2.50[1.48–4.23], *p* = 0.0006) or working conditions (rural or mix vs. urban, OR = 2.99[1.63–5.48], *p* = 0.0004), tobacco exposure ≥30 years (≥30 years vs. never OR = 3.37[1.63–6.96], *p* = 0.0010) were found to increase the risk of CRC. The absence of moderate alcohol consumption while significantly associated with an increased risk of CRC in multivariate analysis, was not confirmed after bootstrapping.

**Table 3:** Associations with the occurrence of CRC (univariate, multivariate conditional logistic regression and after 1000 bootstrapped multivariate conditional logistic regressions) for clinical covariates

Covariates	Model	Univariate^a^			Multivariate^b^			Bootstrapped multivariate analysis^b^			% of bootstrap selection
		HR	95% CI	*p*-value	HR	95% CI	*p*-value	HR	95% CI	*p*-value	
Living	Rural / Mix vs. Urban	2.82	1.96–4.06	*p* < 0.0001	2.50	1.48–4.23	*p* = 0.0006	2.26	1.36–3.75	0.0015	87
Job	Rural / Mix vs. Urban	3.44	2.24–5.29	*p* < 0.0001	2.99	1.63–5.48	*p* = 0.0004	2.93	1.61–5.34	0.0004	87
Red meat consumption	≥3 portions/week vs. <3	1.63	1.01–2.63	*p* = 0.0458							45
Tobacco consumption	Tobacco exposure <30 years vs. never	0.86	0.60–1.25	*p* = 0.4290	1.17	0.70–1.95	0.5439	1.13	0.69–1.84	0.6235	73
	Tobacco exposure ≥30 years vs. never	1.82	1.10–3.03	*p* = 0.0207	3.37	1.63–6.96	0.0010	2.73	1.39–5.39	0.0036	
Alcohol consumption	> 30 g/day now or in the past vs. ≤ 30 g/day	2.62	1.49–4.59	*p* = 0.0008	1.06	0.50–2.22	0.8725				26
	Never vs. ≤ 30 g/day	2.75	1.77–4.27	*p* < 0.0001	3.05	1.69–5.50	0.0002				
Physical activity	<30 vs. ≥ 30 min/day	5.83	3.74–9.07	*p* < 0.0001	6.35	3.70–10.9	*p* < 0.0001	6.73	4.07–11.14	*p* < 0.0001	100

^a^crude values, ^b^adjusted values

### Association between genetic factors and CRC

Hardy Weinberg equilibrium was respected for all the SNPs studied except for the c.388 A > G (rs2306283) in case; their frequencies for all the patient and splitted in case and control are presented in Additional file 1: Table S1. The univariate analysis results for the 15 SNPs are reported in Table 4. No significant association was observed between any of the SNPs (or haplotypes) and CRC in the whole group. Univariate analysis reported significant environment-gene interactions between alcohol*ABCB1 exon26 (alcohol never*exon26: *p* = 0.0356), alcohol*OATP rs2422149 (alcohol >30 g* OATP rs2422149, *p* = 0.0129), meat*UGT1A7 (meat > 3 time/week*UGT1A7, *p* = 0.0363) and meat*UGT1A8 (meat > 3 time/week*UGT1A8, *p* = 0.0153). After multivariate analysis and bootstrapping, only the meat*UGT1A8 (interaction meat > 3 time/week*UGT1A8, adjusted *p* = 0.033; selected in 66% of the bootstraps) and alcohol*ABCB1 exon26 (interaction alcohol never*exon26, adjusted *p* = 0.0004; selected in 90% of the bootstraps) interactions were retained in the final model. Finally, crude analyses were performed to investigate the effect of these interactions in subgroups. In the subgroup of meat-consumers higher than 3 portions/week (*n* = 84), the *UGT1A8* rs1042597-G variant allele was more frequent in cases (allelic risk based on an additive model; OR = 3.39[1.29–8.89], *p* = 0.02951). In the “never alcohol consumption subgroup” (*n* = 125) the ABCB1 exon26 “T” variant was more frequent in cases (allelic risk based on an additive model; OR = 1.89[1.10–3.39], *p* = 0.0257). No adjusted effect of UGT1A8 and ABCB1 exon26 in the mixed subgroup of meat-consumers higher than 3 portions/week + never alcohol consumption due to the too low number of subject in this specific subgroup (*n* = 16).

**Table 4:** Association with the occurrence of CRC (univariate conditional logistic regression) for genetic covariates

Gene	Variant	Model	Univariate		
			HR	95% CI	*p*-value
*UGT1A6*	rs1105879	C vs. A	1.05	0.82 1.35	0.687
*UGT1A7*	rs11692021	T vs. C	0.97	0.76 1.24	0.817
*UGT1A9*	rs2741045	T vs. C	1.00	0.77 1.29	0.970
*UGT2B7*	rs7438135	G vs. A	1.12	0.89 1.40	0.348
*ABCB1*	rs1128503	T vs. C	0.99	0.78 1.25	0.910
	rs2032582	T vs. G	0.96	0.76 1.21	0.727
	rs1045642	T vs. C	1.05	0.84 1.32	0.683
	Haplotype	TTT/other vs. Other/other	1.02	0.72–1.45	0.898
		TTT/TTT vs. Other/other	0.98	0.59–1.65	0.949
*ABCC2*	rs717620	T vs. C	0.98	0.74 1.28	0.864
	rs2273697	G vs. A	1.03	0.77 1.36	0.853
	rs3740066	T vs. C	1.02	0.81 1.29	0.847
*SLCO1B1*	rs4149056	T vs. C	0.82	0.59 1.12	0.213
	rs2306283	G vs. A	1.03	0.83 1.29	0.770
*SLCO2B1*	rs2306168	CC vs CT	1	0.41–2.43	0.994
	rs12422149	AA vs AG	1.02	0.68 1.52	0.932

## Discussion

In this paired case-control study, we investigated associations between CRC and carefully selected allelic variants of genes involved in the intestinal influx and efflux transport, and in the metabolism of xenobiotics, using a candidate gene approach. In addition, this study is probably unique in that we matched cases and controls based on the 3 most important factors known to be associated with colorectal cancer (sex, age, and living location). Using a simple, practical questionnaire setting out known confusion factors (physical activity, dietary habits, alcohol drinking and tobacco smoking behaviors, working/housing environments), we collected descriptive data to neutralize the influence of all these confounding factors on genetic effects. Cases and controls were matched on the same center to accommodate regional variability in diet, environmental arrangements and population density.

The present study showed no influence on CRC of the most relevant SNPs in the genes coding the influx transporters OATP1B1 and OATP2B1. To the best of our knowledge, the potential association of allelic variants of *SLCO1B1* and *SLCO2B1* with CRC has drawn little attention so far. Only one previous study concerned CRC and an influx transporter, and it showed that the *SLCO1B1* c.521 T > C (p.V174A, rs4149056) SNP was statistically associated with CRC [40]. This discrepancy with our results may be due to the differences in ethnicity, as *SLCO1B1* allele frequencies are known to vary markedly between different populations [46, 47]: in Ozhan et al.’s study in the Turkish population, the frequency of rs4149056 was higher than in our French population. Alternatively, it might be due to the nature of the control arm (hospital inpatients with various diagnoses vs. healthy volunteers).

No significant association was observed either between the other investigated SNPs (or haplotypes) and CRC. However a statistical gene*environment interaction was found between meat consumption and the UGT1A8 variant (minor allele frequency 25.7% in our population) as well as between alcohol and the ABCB1 exon 26 variant (minor allele frequency 48.2% in our population): in both cases the CRC risk was gradually increased by the variant allele (additive genetic model). We have no clear explanation for the interaction between ABCB1 exon 26 and absence of alcohol consumption. Many allelic variants of *UGT1A* have been associated with an increased risk for developing sporadic CRC when the consumption of HCA (MeIQx, PhIP, DiMeIQx) and PAH (BaP) is substantial [15]. Even if subgroup analysis shows consistent results in the present study, where UGTs SNPs are influential in red meat consumers, this is not the case in the whole study groups. Such discrepancy can possibly be explained by i) the low proportion of population consuming red meat; and ii) the lack of reliable data concerning cooking methods, doneness preferences.

Cigarette smoke also contains a variety of PAH, HCA and nitrosamine. The CRC risk was reported to increase with cigarette pack-years, smoking duration, smoking intensity, smoking history in the distant past, and younger age at initiation of smoking [45]. A systematic review and meta-analysis by Liang and al. highlighted a stronger association between smoking and rectal than colon cancer [7]. Concordantly, in our cohort, “heavy smokers” were strongly associated with the risk of CRC even if no stratification by cancer site (proximal vs. distal colon vs. rectum) was performed. However, in the subgroup of heavy smokers, no association was found between CRC and UGT polymorphisms. The combined effects of smoking and the genetic variants on colorectal cancer risk may differ by i) metabolism of PAH, HCA and nitrosamine supported too by cytochrome (CYP1A1, CYP1A2, CYP2E1, and CYP2A6), glutathione-S-transferase (GSTM1, GSTT1, and GSTP1) and N–acetyltransferase, ii) levels of carcinogen intake.

In summary, the linkage between low-penetrance allelic variants of UGTs and CRC is probably not powerful enough to be used as a predictive biomarker in non-selected populations. Also UGT1A8 is specifically expressed in the small intestine. In the present study, the interaction gene-environment between UGT1A8 and meat consumption is likely due this localization and might explain this absence of effect for tobacco.

Surprisingly, a lower frequency of CRC was noted in the population living and working in towns, as compared to subjects with purely rural or mixed (rural working / urban living or rural living / urban working) lifestyles. Even if information on the social-economic status and deprivation, which can possibly be confounding factors [48–50], was not collected in this study, our results clearly suggests that a rural environment may be a risk factor of CRC. A particular attention should be paid to this observation, which may be related to overexposure to chemicals, including pesticides, in rural areas. In North-American studies, the gradient between urban and rural populations appears to be cancer-type dependent [51]. In France, the first results of the AGRIculture and CANcer (AGRICAN) cohort study showed an overall lower incidence of cancers among farmers [52]. Conversely, data obtained in UK suggested an increased risk of breast cancer in populations living nearby agricultural areas (including hazardous waste sites containing pesticides) [53]. As a global trend of these studies, it seems that the impact of environmental factors is different between farm workers and people living nearby agricultural areas. The long-term effects of pesticide exposure on health, especially at low doses, have been a matter of intense research. A significant impact on the incidence of fetal malformations, Parkinson’s disease or certain cancer types is now clear [54, 55].

This study confirms that physical activity is protective against CRC. There is abundant epidemiological evidence from prospective studies showing a lower risk of CRC with higher overall levels, frequency and intensity, of physical activity and there is evidence of a dose-response relationship [4, 56]. To promote health, the American Institute of Cancer Research recommends 60 min/day or 30 min/day of moderate or intense physical activity, respectively. Individuals with physical activity along the lines of these recommendations reduce their CRC risk by 24% compared to sedentary populations [42]. It is worth noting that in this study, a statistically significant benefit was already observed for 30 min/day of moderate physical activity.

One of the limitations of this study is that subgroups of patients with confounding factors (lifestyle and environment) were not stratified at baseline.. Moreover, the questionnaire was only a statement of the declarative subjects’ lifestyle at baseline and may thus not be representative of the subjects’ lifestyle over the years prior to enrolment (during which cancer developed in cases), although only minor changes in habits are expected in a population of sixty year-old people.

A second limitation of this study is that it is limited to the French population, and because dietary determinants of CRC may greatly vary across geographic locations, the contribution of low-penetrance genes to the overall risk may vary across populations. A third limitation is that there was no specific selection for control; they were selected in the Clinical Investigation Center from the Official National Healthy Volunteers file.

We made a careful selection of the allelic variants to be tested, using only those with the highest level of recommendation for further research in CRC risk factor (Table 1). Our study highlighted that despite the clinical relevance of allelic variants of UGTs and transporters, their low-penetrance probably weakened their interest as predictive biomarkers in a non-selected population. Furthermore, this study focused on phase II metabolizing enzymes, while there is a growing awareness that interaction between multiple genes play an important role in the risk of common, complex multi-factorial diseases like cancer. The potential influence of allelic variants of phase I enzymes might explain partly the negative results of the present study. The large variability of findings reported so far is not surprising because each genetic variant in the HCA- and PAH-metabolizing pathway plays a minor role in the activation or detoxification of these compounds. Therefore, it is important to combine information from multiple genetic variants to capture the HCA- and PAH-metabolizing pathway (i.e. cytochrome, SULT, GST…) in order to further identify metabolizing risk population, which in turn requires studying very large populations.

In our study, the occurrence of sporadic cancer seems to be more influenced by lifestyle or environment than by a predisposition linked to an allelic variant of low-penetrance. Nevertheless, in a population with a risk factor, the search for allelic variants (as well as the combination with other variants of the metabolism cascade) could be an interesting biomarker.

## Conclusions

In conclusion, the main findings of this large case-control study are the absence of association of CRC with genetic variants of influx transporters, the diet-dependent association with UGT gene variants, and the lower incidence of CRC in the exclusively urban population. Understanding the interaction between modifiable risk factors and genetic susceptibility may support the development of tools for cancer primary prevention strategies.

## Additional file

Additional file 1: Table S1. SNPs studied and their frequencies in our population. This table contains the description and frequencies of the SNP investigated in the study for ABCB1, UGT, MRP2, SLCO1B1 and SLCO1B2 genes. (DOCX 22 kb)

## Abbreviations

CRC: Colorectal cancer
HCA: Heterocyclic amines
LOR: level-of-recommendation
MAF: minor allelic frequency
MRP2: Multidrug Resistance-associated Protein 2
NSAID: Non-steroidal anti-inflammatory Drug
OATPs: Organic anion-transporting polypeptides
PAH: Polycyclic aromatic hydrocarbons
PCR: Polymerase Chain Reaction
P-gp: Permeability-glycoprotein:
QOE: quality-of-evidence
SNPs: Single Nucleotid Polymorphisms
UGTs: UDP-glucuronosyltransferases
