# Supplementary material for: Common variants in glucuronidation enzymes and membrane transporters as potential risk factors for colorectal cancer: a case control study
Source: BMC Cancer. 2017 Dec 28;17:901. doi: 10.1186/s12885-017-3728-0 (PMC5745594; doi:10.1186/s12885-017-3728-0)
Supplement: Additional file 1: Table S1. — SNPs studied and their frequencies in our population. This table contains the description and frequencies of the SNP investigated in the study for ABCB1, UGT, MRP2, SLCO1B1 and SLCO1B2 genes. (DOCX 22 kb) [file 12885_2017_3728_MOESM1_ESM.docx]

Supplemental Table 1: SNPs studied and their frequencies in our population

| **Gene** | **SNPs** | **n** | **Case** | | **Control** | |
| --- | --- | --- | --- | --- | --- | --- |
|  |  |  | **MAF*** | **p HWE** | **MAF*** | **p HWE** |
| ABCB1 | c.1236 C>T (exon 12, rs1128503)  CC  CT  TT | 186  301  100 | 57.5 | 0.477 | 57.2 | 0.405 |
|  | c.2677 G>T (exon 21, rs2032582)  GG  GT  TT | 192  293  102 | 58.2 | 1.000 | 57.2 | 0.552 |
|  | c.3435 C>T (exon 26, rs1045642)  CC  CT  TT | 159  290  138 | 51.2 | 1.000 | 52.4 | 0.725 |
| UGT | 1A6 A>C (rs1105879)  AA  AC  CC | 256  273  58 | 66.3 | 0.036 | 67.4 | 0.597 |
|  | 1A7 T>C (rs11692021)  TT  TC  CC | 238  281  68 | 64.8 | 0.073 | 64.2 | 0.705 |
|  | 1A8 C>G (rs1042597)  CC  CG  GG | 321  230  36 | 73.6 | 0.368 | 74.9 | 0.876 |
|  | 1A9 C>T (rs2741045)  CC  CT  TT | 295  248  44 | 71.4 | 0.317 | 71.3 | 1.000 |
|  | 2B7 A>G (rs7438135)  AA  AG  GG | 142  295  150 | 50.7 | 0.907 | 52.0 | 0.815 |
| MRP2 | c.-24 C>T (rs717620)  CC  CT  TT | 340  214  31 | 76.6 | 0.870 | 76.2 | 1.000 |
|  | c.1249 G>A (rs2273697)  GG  GA  AA | 365  197  25 | 78.7 | 0.600 | 79.2 | 0.477 |
|  | c.3972 C>T (rs3740066)  CC  CT  TT | 224  274  89 | 61.2 | 0.110 | 61.8 | 0.266 |
| SLCO1B1 | c.521 T>C (rs4149056)  TT  TC  CC | 419  155  13 | 85.9 | 0.813 | 83.3 | 1.000 |
|  | c.388 A>G (rs2306283)  AA  AG  GG | 196  270  121 | 56 | 0.005 | 56.8 | 0.634 |
| SLCO2B1 | c.1457C>T (rs2306168)  CC  CT  TT | 567  20  0 | 98.3 | 0.466 | 98.3 | 1.000 |
|  | c.935G>A (rs12422149)  GG  GA  AA | 492  89  6 | 91.3 | 1.000 | 91.5 | 0.451 |

MAF = major allele frequency, HWE=Hardy Weinberg Equilibrium
